# Uterus Transplantation as a Surgical Innovation

**DOI:** 10.1007/s11673-023-10272-5

**Published:** 2023-06-29

**Authors:** Alicia Pérez-Blanco, José-Antonio Seoane, Teresa Aldabo Pallás, Montserrat Nieto-Moro, Rocío Núñez Calonge, Alfonso de la Fuente, Dominique E. Martin

**Affiliations:** 1 Organización Nacional de Trasplantes (ONT), Madrid, Spain; 2 Philosophy, Constitution and Rationality Research Group, Faculty of Law, Universidade da Coruña, A Coruña, Spain; 3 Intensive Care Unit, Hospital Universitario Virgen del Rocío, Sevilla, Spain; 4 Paediatric Critical Care Unit, Hospital Infantil Universitario Niño Jesús, Madrid, Spain; 5 UR International Group, Madrid, Spain; 6 Instituto Europeo de Fertilidad, Madrid, Spain; 7 Geelong Waurn Ponds Campus, Locked Bag 20000, Geelong, VIC 3220 Australia

**Keywords:** Uterus transplantation, Surgical innovation, Medical ethics, Vascular composite allografts, Research ethics

## Abstract

Uterus transplantation (UTx) research has been introduced in several countries, with trials in Sweden and the United States producing successful outcomes. The growing interest in developing UTx trials in other countries, such as Spain, the Netherlands, Japan, and Australia, raises important questions regarding the ethics of surgical innovation research in the field of UTx. This paper examines the current state of UTx in the context of the surgical innovation paradigm and IDEAL framework and discusses the ethical challenges faced by those considering the introduction of new trials. We argue that UTx remains an experimental procedure at a relatively early stage of the IDEAL framework, especially in the context of *de novo* trials, where protocols are likely to deviate from those used previously and where researchers are likely to have limited experience of UTx. We conclude that countries considering the introduction of UTx trials should build on the strengths of the reported outcomes to consolidate the evidence base and shed light on the uncertainties of the procedure. Authorities responsible for the ethical governance of UTx trials are advised to draw on the ethical framework used in the oversight of surgical innovation.

## Introduction

Uterus transplantation (UTx) is a potential treatment to restore reproductive function to women with absolute uterine factor infertility (AUFI)(Richards et al. 2019). An estimated one in five hundred women of childbearing age lack a functional uterus (genetically or acquired) and may experience AUFI (Ngaage et al. 2020; Jones et al. 2021). In contrast to the transplantation of solid organs (e.g., heart, liver, lungs) and like many vascular composite allografts (VCA), UTx may be considered “life-enhancing” rather than “life-saving” (Network n.d.; Johannesson, Wall, et al. 2021a, b, c). It offers some women with AUFI the possibility of experiencing gestation and childbirth (Järvholm et al. 2020), enabling them to create families that would not otherwise be possible (Richards et al. 2019; Testa, Koon, and Johannesson 2017; Riggan et al. 2020).

Following the success of the first trials led by Brännström and colleagues in Sweden, UTx programmes have been introduced in a number of countries over the past decade (Brännström, Belfort, and Ayoubi 2021; Testa et al. 2020). Many report growing acceptance of UTx as a potential treatment for women with AUFI (Jones et al. 2021; Brännström, Kvarnström, and Dahm-Kähler 2020a, b, c; da Graca et al. 2021). As more women seek to undergo UTx, there is evidence of increasing acceptance of the procedure and a shift in ethical discourse about UTx to focus on issues of informed consent from living donors (LD) and transplant recipients, and on equity of access to the procedure (Richards et al. 2019; Ngaage et al. 2020; Kristek et al. 2019). Some commentators, however, argue that “UTx is still in its infancy” (Chmel et al. 2019), noting that it remains an experimental procedure (Brännström, Kvarnström, and Dahm-Kähler 2020a, b, c; Brännström 2019), and as such, should be governed by the existing ethical frameworks for surgical innovation (SI) (Farrell et al. 2020; Flyckt et al. 2018). Growing interest in the development of UTx in countries with established organ transplant systems, such as Spain, Australia, Japan, and the Netherlands raises important questions regarding the ethical implications of new UTx trials and programmes (Bruno and Arora 2020; Kisu et al. 2021; Carmona et al. 2021; Peters et al. 2020). The recognition of UTx as a surgically innovative procedure has significant implications for countries and clinician-researchers when designing and implementing UTx trials. In this paper we explore these implications and argue that ethical scrutiny of proposed UTx trials is essential in order to protect participants and to support free and informed decision-making in a setting of considerable uncertainty and varying practices (da Graca et al. 2021; Balayla 2016; Williams 2016; Hammond-Browning 2019; Daolio et al. 2020; O’Donovan, Williams, and Wilkinson 2019). In particular, we highlight the need for international cooperation that will facilitate consolidation of knowledge and help to support the responsible translation of new innovations in the field such as the use of robotic assisted surgery.

## A Brief History of UTx Trials

The first reported case of human UTx was performed in 2000 in Saudi Arabia, demonstrating partial success with graft survival of ninety-nine days (Fageeh et al. 2002). Since that time, the milestones of success have evolved, with progressive achievements in graft survival and functionality leading eventually to the first live birth following UTx from a living donor (LD), which was reported in Sweden in 2014 (Brännström et al. 2015; Johannesson et al. 2020). In earlier studies, the successful surgical rate has been defined as regular menstruation by recipients following transplantation, with a rate of 78 per cent in the Swedish trial from nine transplants (Brännström et al. 2014, 2015). The largest, still ongoing trial is the Dallas study, which has reported twenty UTx, with twelve births from eleven mothers (birth rate per attempted Utx 55 per cent)(Testa et al. 2020; Johannesson, Testa, et al. 2021a, b, c; York et al. 2021). Up to December 2021, the results of sixty-two UTx cases and twenty-four births have been published in scientific journals (Brännström, Belfort, and Ayoubi 2021). Although many trials have been registered on https://clinicaltrials.gov, at present few have been completed and published results (Brännström et al. 2018).

These trials have sought to address two key scientific challenges—determining optimal venous outflow and immunosuppressive regimes that will maintain uterus grafts ideally for five years, thereby aiming to enable two successful births for recipients (Richards et al. 2019; Brännström et al. 2018; Johannesson, Koon, et al. 2021a, b, c; Ayoubi et al. 2019). However, researchers have also contended with a range of additional challenges in the various trials, with some using grafts from LD, others from deceased donors (DD), and others using both (Testa et al. 2020; Fronek et al. 2021; Flyckt et al. 2016; Ejzenberg et al. 2018). The surgical techniques employed in UTx have also evolved over the past two decades. In many trials there has been a shift from open laparotomy to “minimally invasive surgery” (laparoscopy and robotic assisted laparoscopy) to diminish the invasiveness of the dissections in the LD pelvis during the obtention of vascular pedicles (Brännström, Kvarnström, and Dahm-Kähler 2020a, b, c; Huang et al. 2020; Brännström, Dahm-Kähler, Kvarnström, et al. 2020b, c, a). Trials are increasingly using robotic assisted laparoscopy in an effort to minimize the risks of surgery for LDs while optimizing the quality of vessels pedicles obtained with the donated uterus and hence the vascular connections of the graft (Testa et al. 2020; Carmona et al. 2021; Brännström, Dahm-Kähler, Kvarnström, et al. 2020a, b, c; Brännström, Dahm-Kähler, Ekberg, et al. 2020a, b, c). Significant changes to immunosuppression regimes and the timing of embryo transfer have also been reported; for example, the time to transfer the embryo has shortened to six or three months after transplant when there are no rejection episodes, at which time a transition is made to non-teratogenic immunosuppressants (Brännström et al. 2019; Testa et al. 2018).

As a consequence of this variation in key components of UTx trials, new milestones are frequently reported even as new procedures take considerable time to achieve the successful outcomes noted in other trials. Successes must therefore be carefully evaluated in the context of specific trials. For example, in 2020, twelve births from eleven mothers among twenty transplant recipients (eighteen from LD) were reported in the DUETS trial in the United States (Johannesson, Testa, et al. 2021a, b, c). Transplants performed with DD grafts have notably not yet achieved the success associated with UTx from LDs such as those reported in the Swedish and Dallas trials; the collective reported surgical success rate of DD UTx is 64 per cent, with two live births reported as of December 2021 (Brännström, Belfort, and Ayoubi 2021). Success in trials using minimally invasive surgery also remains mixed. The first fully robotic hysterectomy was performed in China in 2017, with no reported childbirths (Wei et al. 2017). In 2020, a Swedish team reported the first childbirth following UTx, which employed robotic assistance for the vascular dissection but completed the LD hysterectomy with open laparotomy (Brännström, Dahm-Kähler, Kvarnström, et al. 2020a, b, c). The same group performed five hysterectomies with full robotic assistance, obtaining a functioning graft (myometrial flow on postoperative days one and five on doppler ultrasound and menstruation) (Brännström, Dahm-Kähler, Ekberg, et al. 2020a, b, c). In 2020, a Spanish team performed a fully robotic assisted hysterectomy of a LD but has not yet achieved a live birth in this trial (Carmona et al. 2021).

In short, even as the collective experience of UTx research expands, constant innovations within the field mean that data are not always cumulative. The merits of new trials must sometimes be evaluated without the reassurance that more linear development on existing foundations might provide. This is particularly important to consider when evaluating the potential benefits and risks of proposed trials, as reported outcomes for previous trials may not always be applicable in the setting of new innovations.

## Challenges for Countries and Institutions Considering the Introduction of UTx Trials

The variety of cohorts, objectives, and surgical approaches noted in the previous section makes it difficult to establish a robust evidence base for the efficacy and safety of UTx, although this is changing with growing experience in centres in the United States and Europe (Brännström, Belfort, and Ayoubi 2021; Ricci, Bennett, and Falcone 2021). For countries and institutions considering implementing or participating in a first-in-country UTx trial, it may therefore be difficult to access and fully comprehend the available data from previous research in the field due to the limited number of cases reported (often incompletely) and to the heterogeneity of procedures and protocols used in the various studies published since 2000 (Richards et al. 2019; Brännström, Kvarnström, and Dahm-Kähler 2020a, b, c).

Moreover, the continuous evolution of surgical and medical approaches has significant implications for the potential benefits and risks that participants in trials may experience, and hence for decision-making about the design of—and participation in—trials. As noted above, clinician-researchers are now refining surgical techniques and protocols for prevention of graft rejection while minimizing the impact of immunosuppression (Brännström, Belfort, and Ayoubi 2021; Johannesson et al. 2020; Jones et al. 2021). Potential benefits and risks may be strongly influenced by the expertise of the individuals and specifically composed teams involved. Many surgeons have limited experience, for example, with robotic assisted surgery which is associated with a steep learning curve (Brännström, Belfort, and Ayoubi 2021; Dahm-Kähler, Kvarnström, and Brännström 2021). Outcomes of UTx trials involving robotic surgery may thus be particularly influenced by the level of experience within specific trial teams.

An additional challenge is that data from UTx trials may also be limited owing to a reluctance to publish studies and case reports with negative findings, and the difficulty in identifying data from current trials due to a lack of systematic reporting of trials. To overcome this, the International Society of Uterus Transplantation (ISUTx) has established a registry designed to compile data related to transplant procedures and outcomes, which is currently in development (Hammond-Browning and Williams 2020). Johanneson and colleagues from the U.S. Uterus Transplant Consortium have also recently published “guidelines for standardised nomenclature and reporting in UTx” that aim to improve the quality and value of data available to inform oversight and decision-making about trials (Johannesson et al. 2020). Finally, the recency of the field means that there is currently little information about longer term outcomes for trial participants and children created through UTx. Of the twenty-four live births reported after UTx, for example, nineteen children were born pre-term and nine experienced respiratory distress syndrome, making longer follow up essential to evaluate any permanent sequelae that may be associated with specific protocols (Brännström, Belfort, and Ayoubi 2021).

In short, it remains difficult for clinicians, prospective trial participants, and new researchers to predict the probability of complications and success in new trials. For this reason, it is particularly important that the introduction of UTx in new settings be subject to rigorous ethical governance. This requires the recognition of UTx not only as a form of SI, but as a complex experimental procedure.

## UTx as an Experimental Procedure

SI is considered the embryonic stage of surgical research, which may involve

… a novel procedure, a significant modification of a standard technique, a new application of or new indication for an established technique, or an alternative combination of an established technique with another therapeutic modality that was developed and tested for the first time … (Reitsma and Moreno 2002, 793)

SIs often have the primary aim of benefiting specific patients, unlike more routine clinical research, which consists of “a systematic investigation, including development, testing, and evaluation, designed to develop or contribute to a more generalizable knowledge” (Steneck 2007, 40). The initial results of a SI are frequently published in the form of a case report. Once proof of concept and preliminary positive outcomes are established, innovations must be replicated consistently to determine efficacy and safety and subjected to rigorous scientific research methods to evaluate the intervention in a wider population (National Commission for the Protection of Human Subjects of Biomedical and Behavioral Research 1979).

Critics of surgical research and innovation typically focus on the absence of randomized controlled trials (RCTs) and the lack of external oversight (Ergina et al. 2009; Barkun et al. 2009). The five-stage IDEAL framework (see Table 1), established to improve the quality and governance of surgical and other interventional research carried out under an innovation pathway (Khachane et al. 2018) offers a series of recommendations with regard to progressively increasing sample size, methodology, assessment of outcomes, and ethical oversight (McCulloch et al. 2013; Ergina et al. 2013).

**Table 1:** IDEAL stages

	Stage 1 (idea)	Stage 2a (development)	Stage 2b (early dispersion and exploration)	Stage 3 (assessment)	Stage 4 (long-term monitoring)
Number of surgeons	Very few	Few (the innovators)	Many (innovators progressing in their learning curves)	Many, early majority	All eligible
Number of patients	Single to few	10s	100s	100s+	100s+
Ethical oversight	Informed consent only Not required in life-threatening conditions	Register protocols and local ethical approvals	Standard research ethics approvals	Standard research ethics approvals	Informed consent only
Measurement of outcomes	Case reports	Prospective development studies	Feasibility RCT	RCT or alternative designs	Registry, audit
Purpose/ Outcomes	Proof of concept in humans	Safety, development of technical details of procedure	Reproduction of procedure to improve efficiency (e.g. shorter operative times) and effectiveness. Short-term outcomes.	Procedure is part of many surgeons’ practice, but still performed on selected patients Cost-effectiveness Specific long- term outcomes	Procedure is routine practice and permits long-term monitoring of rare events

The field of UTx is currently at an IDEAL stage 2a–2b (Barkun et al. 2009). Small prospective studies are being conducted and the number of participants currently enrolled remains small. Most trials are recruiting pairs of prospective LDs and recipients with AUFI; high-volume transplant centres that have already registered trials intend to enrol between five and a maximum of twenty participants (see www.clinicaltrials.gov). SI in the field of UTx has not yet progressed to the stage of conducting coordinated trials in which a specific, consistent protocol is refined over time with increasing experience and evidence, as has occurred, for example, in relation to laparoscopic LD nephrectomy. The latter was first performed in humans by Ratner in 1995 (Manikandan and Sundaram 2006) and then repeated as a minimally invasive alternative to an open surgery by Flowers (70 LDs) (Fonouni et al. 2014), and Fabrizio (110 LDs) (Fabrizio et al. 1999). The technique was subsequently refined and made more reproducible by Jacobs’ three-year study involving 320 procedures (Jacobs et al. 2000). However, as Ramani et al. report, the risks and complications of LD hysterectomy in the UTx have not yet been analysed and assessed to the same evidentiary standards as LD hepatectomy or nephrectomy (Testa et al. 2020).

*De novo* UTx trials across different centres are expanding rapidly worldwide, enabled by Brännström and his team in sharing the pioneers’ experience. Owing to the presence of significant variations between many of them, however, most new trials cannot claim to be building on current international experience in the field, especially those involving DD UTx or the novel robotic-assisted laparoscopic approach. They must therefore be evaluated and ethically managed on the basis of their own merits and limitations as early-stage innovations (Matoba et al. 2021). As recommended by the IDEAL framework (Ergina et al. 2013), the next stage in the development of UTx would ideally involve international trials of specific procedures, such as UTx from deceased donors or LD UTx involving robotic surgery, with standardized medical and surgical protocols. International trials would in theory permit the inclusion of larger cohorts of participants and clinical teams with more consistent experience in UTx (Ergina et al. 2009; 2013; Barkun et al. 2009; McCulloch et al. 2013). However, there are several potential barriers to such trials, including differences in institutional capacity and current professional expertise, as well as potential differences in regulations governing assisted fertility treatments and in deceased donation programmes, which limit the ability to implement standardized protocols and participant cohorts. For example, centres with the ability to perform robotic assisted surgery would likely be reluctant to revert to open hysterectomy with its higher burdens for LDs (Johannesson, Koon, et al. 2021a, b, c; Dahm-Kähler, Kvarnström, and Brännström 2021). Conversely, the steep learning curve of robotic surgery may limit the capacity of many centres to introduce this technique. Variations within research teams, clinical settings, and trial cohort characteristics may notably impact the potential benefits and risks of particular protocols in specific contexts, with implications for the ethical justification of trial protocols. The most feasible protocol in one country may not be considered best practice in another country, such that achieving consistency between countries may prove difficult.

Nevertheless, greater standardization of protocols and more consistent definition of variables and outcome measures may help to support comparative analysis of trials, as recommended by Johanneson et al. (Johannesson et al. 2020). A cooperative rather than competitive approach to UTx research will help to facilitate coordination of research activities between different programmes and support production and communication of more generalizable knowledge that will be beneficial for all. The ISUTx was founded with this mission in 2016; assisting researchers in developing and implementing new trials in a coordinated, collaborative manner and in developing “consensus guidelines for uterus transplantation” (Flyckt et al. 2020). In 2019, the ISUTx announced the creation of a registry to collect data concerning LDs, recipients, transplantation surgery, postoperative complications, immunosuppression, and live births (Brännström 2019) to meet the need for an open international registry to help navigate the transition from experimental procedure to clinical treatment in accordance with the IDEAL framework (Ricci, Bennett, and Falcone 2021; Reitsma and Moreno 2002).

Despite the benefits of adoption of consistent nomenclature, outcome reporting, and contributions to an international registry, researchers may require encouragement to implement these steps, especially if doing so creates additional administrative burdens in the conduct of trials. In addition to designing tools and data collection systems that are easily accessible, adherence to international recommendations may be increased by strategies such as a requirement to report trial data to the ISUTx registry in order for publication of research to be considered. A consistent expectation in the field that standardized nomenclature and outcome measures will be used when reporting results should also encourage compliance.

## Ethical Governance of Surgical Innovation and Implications for UTx

Lack of independent ethical oversight exposes patients to risks and jeopardizes the credibility of reported outcomes, particularly when innovations are introduced without formal evaluation (Glazier 2016; Fonouni et al. 2014; Broekman, Carrière, and Bredenoord 2016; Ceelen 2014). To avoid this, all SIs, including trials of UTx, should be subject to the fundamental principles of human research ethics, including independent oversight by trained and accredited human research ethics committees (Reitsma and Moreno 2006; Biffl et al. 2008).

Non-specialist human research ethics committees may not be experienced in the review of surgical research, and alternative governance processes may be needed to facilitate the timely and effective introduction and evaluation of innovations. The call for ethics oversight mechanisms in relation to SI is well attested in the literature (Biffl et al. 2008; Broekman, Carrière, and Bredenoord 2016; McKneally 1999; Morreim, Mack, and Sade 2006; Johnson and Rogers 2012; Karpowicz, Bell, and Racine 2016), although there is some debate regarding the composition of such bodies, the extent of their oversight responsibilities, and the type of studies they should oversee. Nevertheless, their core principles are largely standard: well-being of trial participants, prevention of avoidable harm, management of potential conflicts of interest (COI) on the part of investigators, and objective assessment and disclosure of outcomes and complications Table 2.

**Table 2:** Key considerations for ethical review of UTx trials

Individual and institutional capacity
Key requirements to be satisfied before planning a trial: Clinician-researcher experience in transplant surgery. Field strength: previous surgery in animal models. Institutional stability: background of close work of health professionals from all departments involved.
Favourable risk-benefit balance
Avoid causing harm to participants or risk minimization. Risks balanced with the expected benefits for both parties (living donors and recipients) and the current rate of live birth per attempted UTx.
Enhanced informed consent process (I) Information
Information should be explained in an intelligible way for the participant. Detailed disclosure of the innovative nature of the procedure.
Enhanced informed consent process (II) Consent
Consent should be updated in each of the UTx phrases. Consent withdrawal should be facilitated.
Enhanced informed consent process (III) Voluntariness
Exploitation or undue influence should be prevented.
Conflict of interests
Potential conflicts of interests should be routinely disclosed to all stakeholders and carefully managed where these are unavoidable Systematic reporting of trial results.

### Individual and Institutional Capacity to Safely Implement an Innovative Procedure

In 2000, Moore proposed three requirements for the ethical introduction of SIs: firstly, surgeons should have solid laboratory experience of performing the new operation on large animals and cadavers; secondly, researchers should have substantial experience of previous collaboration in the field; and thirdly, institutions should have the stability to deal with any problems or challenges that may arise (Moore 2000). Moore’s recommendations of institutional capacity and surgical expertise are now well established and can be found in the policies and practices of bodies such as the American Society for Reproductive Medicine and in the ISUTx.^1^ Pioneers such as Brännström and Ayoubi have also reiterated the need for training with non-human animals in the pre-clinical phase of UTx research (Brännström 2019; Ayoubi et al. 2019).

### Risks and Benefits

The ethical conduct of all research should be guided by the harm minimization principle. Research should minimize risks to participants, such that only risks that are strictly necessary to attain the objectives are tolerated, and only when these are balanced by the expected benefits. For UTx trials, as in trials of other VCAs, the substantive risks and burdens associated with transplant surgery and immunosuppression for the recipient and the LD require particularly careful consideration given that the procedure is not a lifesaving one.

UTx is also distinct in terms of its transitory nature (Bruno and Arora 2020): the procedure is performed with the expectation that once reproductive goals have been achieved, uterine grafts will be removed in order to avoid the long-term risks associated with immunosuppression (Testa, Koon, and Johannesson 2017). It is considered that living donation should only be performed “when the aggregate benefits to the donor–recipient pair . . . outweigh the risks to the donor–recipient pair” (Pruett et al. 2006, 1386). Even more challenging is the justification of risks incurred by LDs, given the existence of a successful DD UTx alternative (Ejzenberg et al. 2018). Nevertheless, individuals are often permitted to assume similar burdens and risks for similar objectives in different settings. For example, living kidney donation is permitted even where DD transplantation and dialysis provide viable if not optimal alternatives for patients with kidney failure (Reese, Boudville, and Garg 2015; Wiedebusch et al. 2009), gestational surrogacy and onerous assisted reproductive treatments are permitted to help individuals achieve their procreative goals, and many healthy volunteers are permitted to participate in potentially hazardous clinical trial research.

There are many substantive risks and potential benefits for UTx recipients and for LDs (Järvholm et al. 2019; 2020); relevant risks and potential benefits must be carefully assessed in the specific context of particular trial protocols given the many factors outlined above which may influence outcomes as innovative procedures are introduced. It is also important that information about risks and potential benefits be communicated to prospective trial participants in a manner that supports autonomy and valid consent. Framing bias, for example, may result from consultation of the literature in which the emphasis is often placed on proportionate success rather than substantial failure rates. Both clinician-researchers and patients who are potential trial participants may be susceptible to framing biases (Perneger and Agoritsas 2011).

### Consent

Consent is always the foremost consideration for ethical participation in clinical trials; it is complicated in the context of SIs by the limitations of knowledge and the fact that, in contrast to typical research, innovation trials are often primarily intended to be therapeutic for the participants. Thus, while therapeutic misconception may not pose as great a concern (Miller, Rosenstein, and DeRenzo 1996), transplant recipient participants are still at risk of compromised decision-making and exploitation owing to their strong personal interest in the outcomes of the procedure. Emphasis on the potential positive outcomes of UTx and the lack of alternative treatments may distract trial participants from the limitations of the evidence base for the procedure. Furthermore, since LD participants may view their role primarily as that of a therapeutic donor rather than an experimental trial subject, it is important that both recipients and LDs (where applicable) are supported in making informed decisions about their participation (Testa et al. 2020; Järvholm et al. 2018; Petrini et al. 2017).

Disclosure of the limitations of knowledge regarding the possible risks and benefits of the procedure is crucial in order to ensure transparency and valid consent (Allyse et al. 2018). Researchers may differ significantly from participants regarding their view of what constitutes essential information (Ceelen 2014; Järvholm et al. 2018) and must therefore pay careful attention to what and how much information is needed for consent to be valid. They should clarify to participants the limitations of current knowledge of potential benefits and risks and their implications, including the fact that the evidence available is drawn from a heterogeneous set of small-scale trials.

While knowledge generation may be a secondary concern in SI research, the presence of research-related goals can lead to COI on the part of clinician-researchers that should be disclosed to participants in trials (see below).

Some candidates for UTx may be vulnerable to exploitation or undue influence because of familial or societal values and norms with regard to infertility or as a result of psychiatric disorders associated with AUFI (Saso et al. 2014). Prospective LDs may also be subject to pressures or coercion, as has been observed in relation to living donation of other organs (Elliott 1995; Abdeldayem et al. 2014; Valapour et al. 2011). Knowing, for example, that recipients depend on them for the chance to achieve their reproductive goals through UTx can make it difficult for donors to withhold or withdraw consent.

Specific considerations also apply in the context of DD UTx, where explicit consent for uterus retrieval should be obtained from those responsible for making or confirming a decision on behalf of a potential DD (Caplan et al. 2007).

### Conflicts of Interest

Innovators may be motivated by a range of potentially conflicting interests which may influence decision-making to the detriment of trial participants and outcomes. These may include the desire to shed light on scientific enigmas, to earn public acclaim, for example (Glazier 2016; Moore 2000; Mcdonald et al. 2010). COIs may also create unconscious bias in researchers, which may be greater in the case of a novel procedure with possible benefits for patients. Subsuming their scientific role into that of a clinical care provider, some clinician-researchers may overlook the still experimental nature of UTx and the fact that recipients and LD are not just patients but also research participants (Moore 2000; Morgenstern 2008). The language of published UTx research often obscures this distinction in emphasizing clinical outcomes for patients rather than research participants. Clinician-researchers, like patient-participants, may be susceptible to therapeutic misconception as they strive to manage their “competing loyalties” to research and patient care (Petrini et al. 2017). This can also lead to bias when recruiting participants and communicating information that may inform decision-making about participation in trials (Petrini et al. 2017).

The transparent publication of trial results and consistent reporting of outcomes, as encouraged by ISUTx, will help to provide prospective trial participants and clinician-researchers with a wider evidence base for their decision-making and facilitate recognition of potential bias.

## Conclusions and Recommendations

UTx is still in the early stages of the IDEAL framework and has not yet completed the phases required to be considered a standard treatment for AUFI. The rapid acceleration of UTx research may soon resolve some of the uncertainties and concerns presented here. However, despite the growing number of case reports, heterogeneity of experience means the evidence base remains limited.

When new trials are considered in any country, it is vital that researchers work closely with those responsible for the ethical oversight of donation and transplant activities and the ethical conduct of human research. When decisions about initiating or expanding trials of UTx are made, they should be informed by existing experience and expertise, both nationally and internationally, and the specific implications of procedures that fall within an innovation pathway rather than a classic research paradigm.

Finally, all those involved in UTx trials should strive to make use of standardized nomenclature and outcome measures and to ensure that all data are reported to relevant registries to support further advances in the field.

## Footnotes

1 https://tts.org/isutx-home
